# ApoE isoform-dependent changes in hippocampal synaptic function

**DOI:** 10.1186/1750-1326-4-21

**Published:** 2009-05-27

**Authors:** Kimberly M Korwek, Justin H Trotter, Mary Jo LaDu, Patrick M Sullivan, Edwin J Weeber

**Affiliations:** 1 Neuroscience Graduate Program, Vanderbilt University, Nashville, Tennessee 37232, USA; 2 Department of Anatomy and Cell Biology, University of Illinois at Chicago, Chicago Illinois 60612, USA; 3 Department of Medicine, Centers for Aging/Geriatric Research Education and Clinical Center, Durham Veteran Affairs Medical Center, Duke University Medical Center, Durham, North Carolina 27710, USA; 4 Department of Molecular Pharmacology and Physiology, Johnnie B Byrd Sr Alzheimer's Center & Research Institute, University of South Florida Tampa, Florida 33612, USA

## Abstract

The lipoprotein receptor system in the hippocampus is intimately involved in the modulation of synaptic transmission and plasticity. The association of specific apoE isoform expression with human neurodegenerative disorders has focused attention on the role of these apoE isoforms in lipoprotein receptor-dependent synaptic modulation. In the present study, we used the apoE2, apoE3 and apoE4 targeted replacement (TR) mice along with recombinant human apoE isoforms to determine the role of apoE isoforms in hippocampus area CA1 synaptic function. While synaptic transmission is unaffected by apoE isoform, long-term potentiation (LTP) is significantly enhanced in apoE4 TR mice versus apoE2 TR mice. ApoE isoform-dependent differences in LTP induction require NMDA-receptor function, and apoE isoform expression alters activation of both ERK and JNK signal transduction. Acute application of specific apoE isoforms also alters LTP induction while decreasing NMDA-receptor mediated field potentials. Furthermore, acute apoE isoform application does not have the same effects on ERK and JNK activation. These findings demonstrate specific, isoform-dependent effects of human apoE isoforms on adult hippocampus synaptic plasticity and highlight mechanistic differences between chronic apoE isoform expression and acute apoE isoform exposure.

## Introduction

More than a decade ago, the allelic variation of apolipoprotein E (apoE) was associated with an altered risk of Alzheimer's disease (AD) development [1,2]. The human population maintains three commonly occurring apoE isoforms that differ at two amino acid positions: apoE2 (Cys^112^, Cys^158^), apoE3 (Cys^112^, Arg^158^), and apoE4 (Arg^112^, Arg^158^). The apoE3 allele is maintained at an allele frequency of approximately 78% in populations of European descent and is described as having no effect on AD risk. ApoE4, with an allele frequency of 14% in these populations, is linked to an increased risk of developing sporadic AD as well as a decreased age of onset compared to inherited apoE3. In contrast, apoE2 expression decreases disease risk compared to apoE3 [1,3].

In the CNS, apoE binds to the seven identified mammalian members of the highly conserved low-density lipoprotein receptor (LDLR) family [4]. Research in the last decade has established that the LDLR family is intimately involved in neuronal signal transduction, modulation of ligand-gated ion channels, and control of neurite outgrowth, synapse formation and neuronal migration (for review see [5]). Of particular interest is the association of apoE with two highly expressed members of the LDLR family: apolipoprotein E receptor 2 (apoER2) and the very low density lipoprotein receptor (VLDLR). Through the experimental use of reelin, an apoER2 and VLDLR ligand, these receptors have been linked to several signal transduction pathways that play a role in synaptic maturation and NMDA receptor modulation in the adult hippocampus. Furthermore, disruption of reelin binding or apoER2 and VLDLR function through deletion or mutation results in learning and memory defects [6-10]. While apoE also associates with these important signaling receptors, relatively little is known about apoE signaling in the CNS in general, or how apoE isoforms may specifically affect synaptic function.

The targeted replacement apoE isoform-expressing mice (apoE TR) are a valuable tool for understanding the role of apoE in memory formation and synaptic function. These mice express one of the three human apoE isoforms under the control of the endogenous murine promoter [11]. The high degree of conservation between murine and human apoE receptors allows for these mice to be used as a general mammalian model for direct comparison of the actions of apoE isoforms [12,13]. Previous work has shown that apoE4 TR mice have impaired spatial memory retention during tests that apoE3 TR, apoE-deficient, and murine apoE-expressing animals were able to perform [14]. Studies of the electrophysiologic response to hippocampal perforant path stimulation revealed tha LTP induction in apoE3 TR mice was equivalent to the wild-type controls, but both apoE2 TR and apoE-deficient animals show significant reduction in LTP induction with further reduction observed in apoE4 TR mice [15]. An investigation of a similar line of apoE targeted replacement mice reveals an age-dependent enhancement of CA1 LTP in young apoE4 TR animals compared to wild-type controls [16]. While these data suggest that the actions of apoE isoforms can alter synaptic plasticity, an in-depth study of hippocampus area CA1 synaptic function using all of the available apoE TR mice is unfortunately absent.

Work from our group and others suggest that apoE has similar signaling capabilities as the extracellular matrix protein reelin and can modulate synaptic function in an isoform-dependent manner. The present study determines apoE necessity and isoform-dependent changes of synaptic transmission and plasticity in area CA1 of the adult hippocampus in apoE TR mice. We reveal differences in the effects of apoE-isoforms under chronic or acute exposure conditions and also explore the possible mechanisms of hippocampal apoE isoform-dependent signaling using both electrophysiological and biochemical techniques.

## Results

### LTP induction in apoE TR animals is isoform-dependent

The extracellular matrix protein reelin signals through both apoER2 and VLDLR; these receptors also bind apoE. However, unlike reelin-deficient mice, apoE TR and apoE-deficient animals develop normally without gross pathological changes to brain organization suggesting that apoE signaling or apoE isoform expression are not involved in neuronal migration during development. This has clear advantages when examining parameters of synaptic function in apoE TR and apoE deficient mice. We find that hippocampal slices from 3–5 month-old animals are equivalent across genotypes and are identical to wild-type hippocampi on a gross anatomical level.

**Figure 1:**
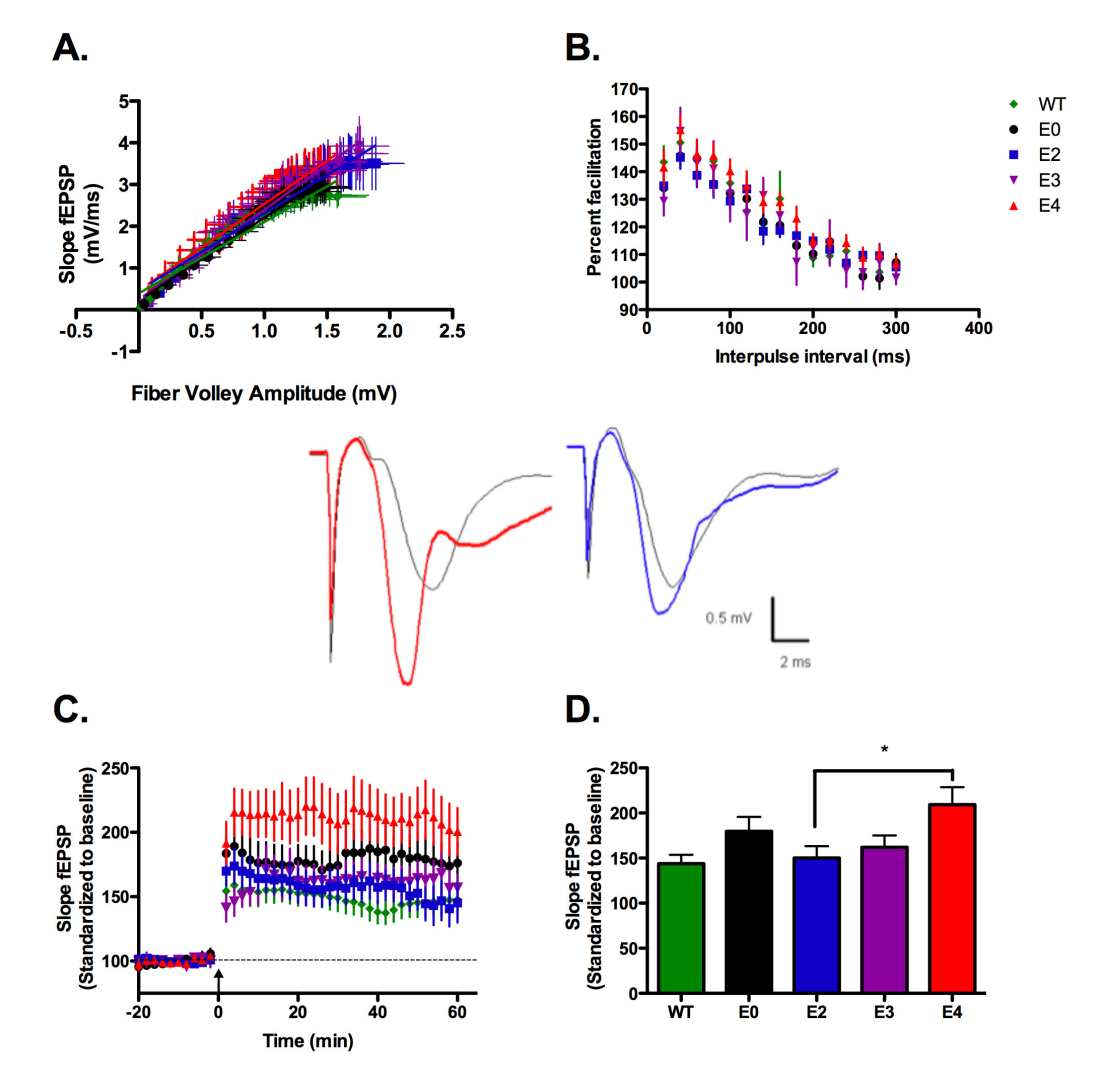
**ApoE4 TR animals show increased LTP induction without changes in synaptic transmission**. A) Input-output curve generated from the slope fEPSP versus fiber volley amplitude measured at increasing stimulus intensities. B) Paired pulse facilitation. Second stimuli delivered at 20 ms intervals from 20 to 300 ms from first stimuli. Percent facilitation of fEPSP slope of second response as percentage of first response. C) Long-term potentation induced by 5 trains of theta-burst stimulation (arrow). Expressed as slope of fEPSP, standardized to the first 20 minutes of recording. Representative traces 5 minutes before (black lines) and 40 minutes after (colored lines) stimulation for apoE4 (red) and apoE2 TR (blue). Scale: 0.5 mV, 2 ms. D) Average potentiation of last 20 minutes of recording. C57BL/6J = WT, green diamond, n = 12; apoE-deficient = E0, Black circle, n = 12; apoE2 TR = E2, blue square, n = 17; apoE3 TR = E3, purple inverted triangle, n = 6; apoE4 TR = E4, red triangle, n = 14. Data expressed as mean ± SEM. *p < 0.05, ANOVA with Bonferroni's posttest.

Supporting the lack of structural changes, hippocampus area CA1 synaptic transmission does not vary significantly with apoE isoform expression (figure 1A). These data ensure an important parameter in plasticity studies: that specific electrical 'input' elicits an equivalent synaptic response 'output' regardless of genotype. Thus, subsequent variation in measured plasticity can be attributed to apoE isoform rather than changes in CA1 synaptic connectivity. Determination of short-term plasticity using paired-pulse facilitation revealed typical percent facilitation between apoE TR, apoE-deficient and wild-type mice (C57BL/6J) (figure 1B).

High frequency stimulation of Schaeffer collateral synapses using a standard theta burst stimulation protocol induces robust potentiation [17,18]. Using five trains of theta burst stimulation, we successfully induced potentiation in wild-type, apoE-deficient, and apoE TR mice. The magnitude of potentiation, however, was dramatically different between apoE isoforms (figure 1C). Immediately following stimulation and persisting for the duration of the experiment, apoE4 TR mice had increased LTP induction (figure 1C). Potentiation in apoE4 TR mice was significantly increased from that seen in the apoE2 TR (figure 1D; ANOVA, p = 0.0198). LTP induction in the apoE-deficient mice was intermediate between apoE2 TR and apoE4 TR, but this trend was not significant. There was no significant difference in the magnitude of LTP induction between wild-type and apoE-deficient animals. Interestingly, the increased CA1 LTP in the apoE4 TR is contradictory to perforant path LTP in the same animals [15,19], suggesting a differential role for apoE in perforant path and CA1 plasticity.

**Figure 2:**
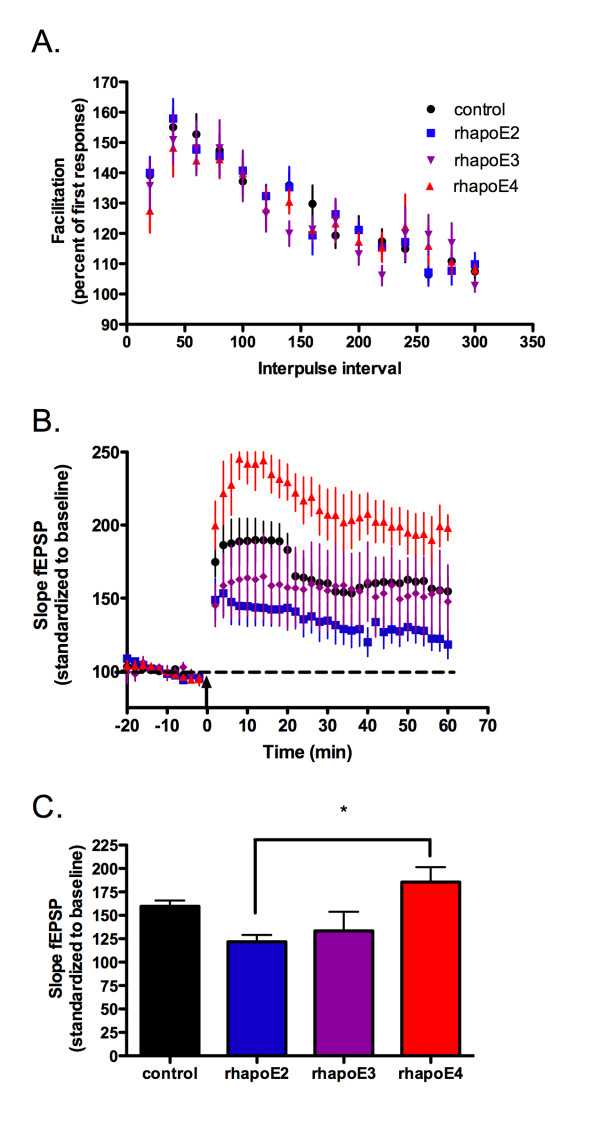
**Acute recombinant apoE isoform application recapitulates alterations in LTP induction**. Application of 100 nM recombinant human apoE isoforms 5 min prior to start of recording. A) Paired pulse facilitation. Second stimuli delivered at 20 ms intervals from 20 to 300 ms from first stimuli. Percent facilitation of fEPSP slope of second response as percentage of first response. B) Long-term potentiation induced by 5 trains of theta-burst stimulation (arrow). Expressed as slope of fEPSP, standardized to the first 20 minutes of recording. C) Average potentiation of last 20 minutes of recording. control = black circle, n = 8; rhapoE2 = blue square n = 8; rhapoE3 = purple inverted triangle, n = 7; rhapoE4 = red triangle, n = 6. Data expressed as mean ± SEM. *p < 0.05, ANOVA with Bonferroni's posttest.

We hypothesize that apoE isoforms differentially act as ligands to a set of lipoprotein receptors that have the ability to modulate synaptic activity. However, the changes in the amount of plasticity between specific apoE isoforms may be due to intrinsic properties of the apoE TR mouse or the production of compensatory protein expression during chronic apoE isoform expression. To test for isoform-dependent signaling differences that could account for our electrophysiology results in the TR mice, we prepared hippocampal slices from apoE-deficient mice and perfused with 100 nM of human recombinant E2, E3 and E4 (rhapoE) prior to theta burst stimulation. ApoE-deficient mice were chosen to insure that the presence of endogenous murine apoE did not interfere with the applied rhapoE. Perfusion of rhapoE2, rhapoE3 or rhapoE4 has no effect on overall baseline synaptic transmission (data not shown) or short-term plasticity (figure 2A). However, there is an isoform-dependent change in synaptic plasticity following theta burst stimulation (figure 2B,C). Interestingly, the change in plasticity with specific isoform perfusion follows a trend similar to that measured in the corresponding apoE isoform TR mouse; both apoE4 TR animals and slices treated with rhapoE4 demonstrate significantly increased LTP induction over apoE2 TR mice or treatment with rhapoE2, respectively (rhapoE: ANOVA, p = 0.0112). This suggests that differences in synaptic plasticity are not developmentally-dependent and that apoE acts as an isoform-specific signaling molecule in the adult hippocampus.

### Effect of apoE isoform on LTP is NMDAR dependent

**Figure 3:**
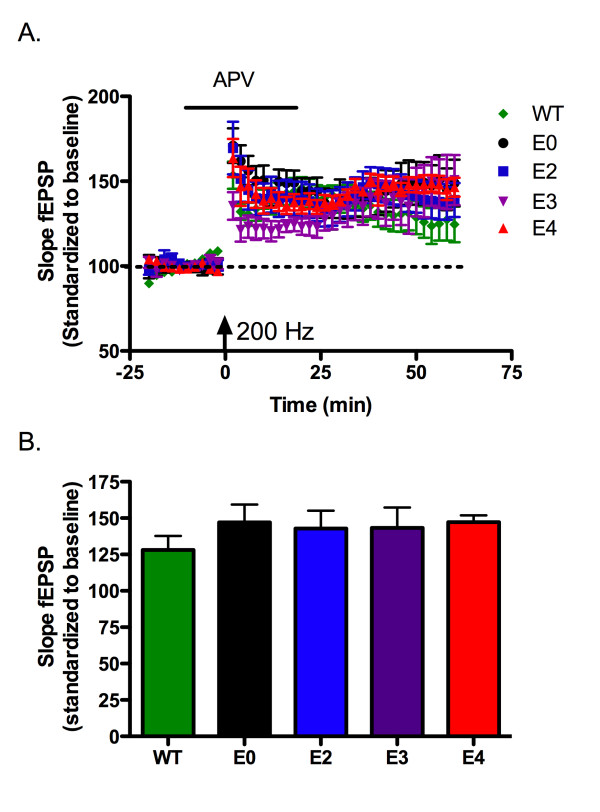
**NMDA receptor-independent LTP is not affected by apoE isoform**. A) Long term potentiation induced by 2 trains of 200 Hz stimulation (arrow). Application of 100 μM APV for 5 minutes before and 20 minutes after 200 Hz stimulation. Expressed as slope of fEPSP, standardized to the first 20 minutes of recording. B) Average potentiation of last 20 minutes of recording. C57BL/6J = WT, green diamond, n = 5; apoE-deficient = E0, black circle n = 8; apoE2 TR = E2, blue square, n = 8; apoE3 TR = E3, purple inverted triangle, n = 7; apoE4 TR = E4, red triangle, n = 6. Data expressed as mean ± SEM.

The NMDA receptor is intimately associated with many forms of synaptic plasticity and long-term potentiation, including theta-burst-induced LTP [18,20]. In addition, signal transduction via apoE receptors is linked to NMDA receptor maturation [21,22], increased NMDA receptor currents [23], and activation of signaling pathways that involve NMDA receptor function [9,23,24]. These results lead us to hypothesize that the observed alterations in LTP may be due to apoE isoform-specific changes in NMDA receptor function. Thus, we induced NMDA-receptor independent LTP by delivering two one-second trains of 200 Hz stimulation concurrent with application of the NMDA receptor antagonist APV (100 μM). Long-lasting potentiation was induced in wild-type, apoE-deficient, and apoE TR animals; however, we eliminated apoE isoform-dependent alterations of LTP induction (figure 3A–B).

The normalization of NMDAR-independent LTP induction suggests that NMDARs are being modified by both the presence of specific apoE isoforms in the TR mice and by exogenous application of apoE isoforms. This can occur through direct changes in NMDAR function indicated by changes in phosphorylation and subsequent modulation of NMDAR conductance. We took advantage of the acute apoE-isoform application strategy to investigate the ability of apoE isoforms to change CA1 NMDA receptor function over time.

**Figure 4:**
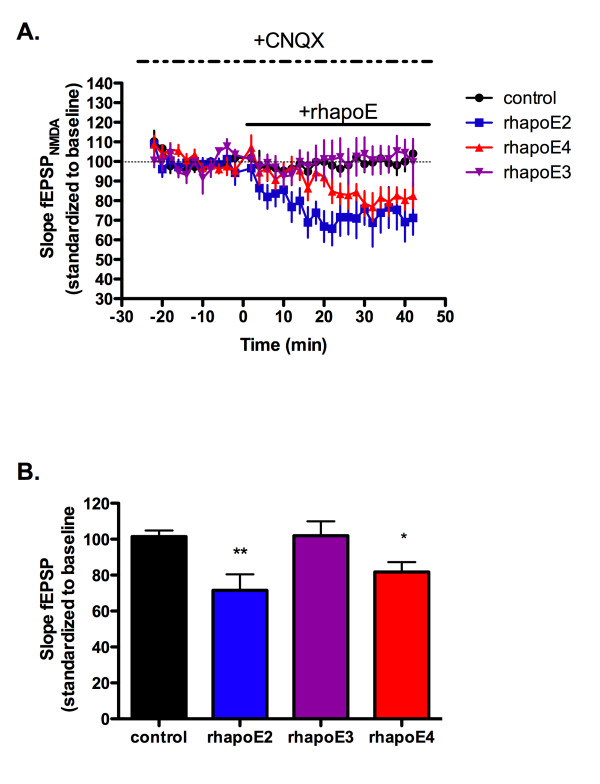
**ApoE isoforms alter NMDA receptor-dependent field potentials**. A) Synaptic transmission in the presence of 20 μM CNQX (dashed line) and 100 nM human apoE (solid line). Expressed as slope of fEPSP, standardized to the first 20 minutes of recording. B) Average potentiation of last 20 minutes of recording. control = black circle, n = 8; rhapoE2 = blue square, n = 8; rhapoE3 = purple inverted triangle, n = 7; rhapoE4 = red triangle, n = 6. Data expressed as mean ± SEM. *p < 0.05, **p < 0.01, ANOVA with Bonferroni's posttest.

NMDA receptor field potentials were isolated by application of 20 μM of the AMPA receptor antagonist CNQX followed by application of 100 nM of rhapoE2, rhapoE3 or rhapoE4. While we hypothesized that the enhanced LTP seen in the presence of apoE4 was related to increased NMDA receptor currents similar to the effect of reelin application, we found that application of either rhapoE2 or rhapoE4 significantly reduced NMDA field potentials from control levels (figure 4A–B). Interestingly, there was no effect on NMDAR field potentials with the application of rhapoE3. Although these results suggest a specific change to NMDA receptors, there is also the possibility that apoE isoforms can alter apoE receptors known to influence NMDA receptors in the hippocampus, such as apoER2.

### ApoE isoforms do not alter apoER2 expression levels

**Figure 5:**
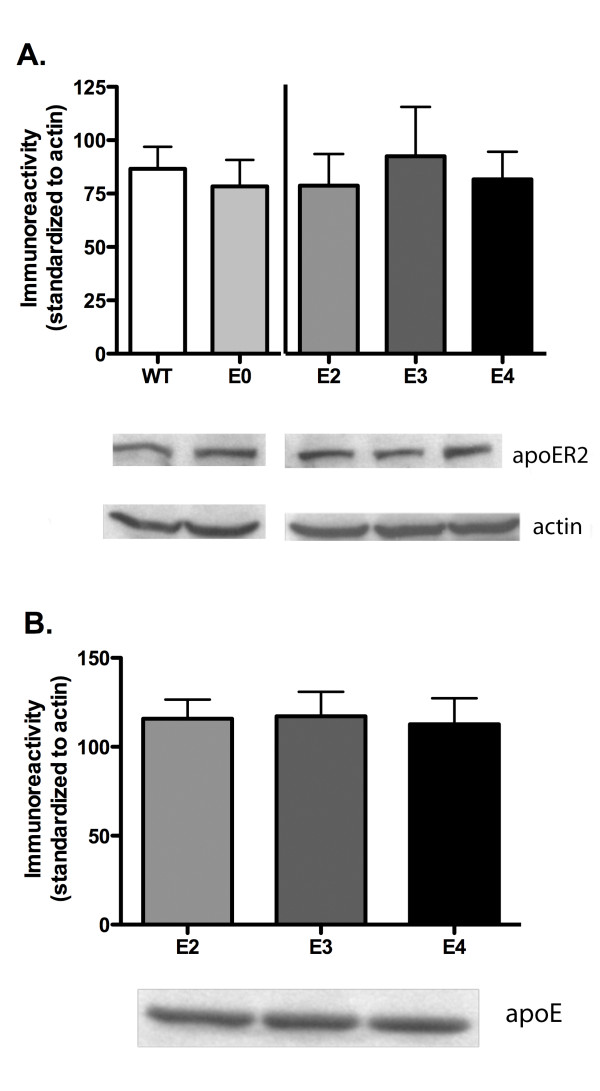
**ApoE isoform expression does not affect apoER2 expression levels**. Representative western blots showing levels of apoE (A) and apoER2 (B) immunoreactivity in whole hippocampus of aged animals. Quantification of immunoreactivity standardized to actin (n = 8, n = 5 for apoE3 TR). C57BL/6J (WT, white), apoE-deficient (E0, light grey), apoE2 TR (E2, medium grey), apoE3 TR (E3, dark grey), apoE4 TR (E4, black). Data expressed as mean ± SEM.

Alterations in apoER2 expression and decreased reelin expression can adversely affect spatial memory [9,25]. Furthermore, changes in reelin concentration and expression can alter LTP strength and induction as well as NMDAR conductance [21,23]. This suggests that differences in receptor expression and concentration as well as changes to lipoprotein receptor ligands may affect synaptic transmission or learning and memory. Therefore, we probed for changes in overall expression levels of the main apoE receptor in the brain, apoER2. Isolated hippocampus tissue was probed for apoER2 expression using western blot analysis. Figure 5A indicates that apoE2, apoE3 and apoE4-expressing animals exhibit no alterations in total apoER2 expression in the hippocampus (ANOVA, p = 0.8269); apoE deficient and murine apoE-expressing animals are also equivalent in receptor expression (t-test, p = 0.6175). Recent studies by Riddell *et al*. [26] suggest a differential apoE isoform expression in whole hippocampus or frontal cortex homogenates. However, our results obtained with slightly different conditions show that targeted replacement did not alter overall apoE expression levels in the hippocampus (figure 5B, ANOVA, p = 0.6912).

### Role of apoE isoform in NMDAR phosphorylation

Tyrosine phosphorylation of NMDA-receptor subunits has been shown to influence NMDA receptor trafficking and assembly [27,28]. This tyrosine phosphorylation, mediated by Src-family kinases such as Fyn, plays an integral role in hippocampal synaptic plasticity and LTP [29,30]. With the identification of reduced NMDA receptor-dependent field potentials with rhapoE2 and rhapoE4, we hypothesized that apoE may be altering the state of NMDA-receptor subunit tyrosine phosphorylation.

**Figure 6:**
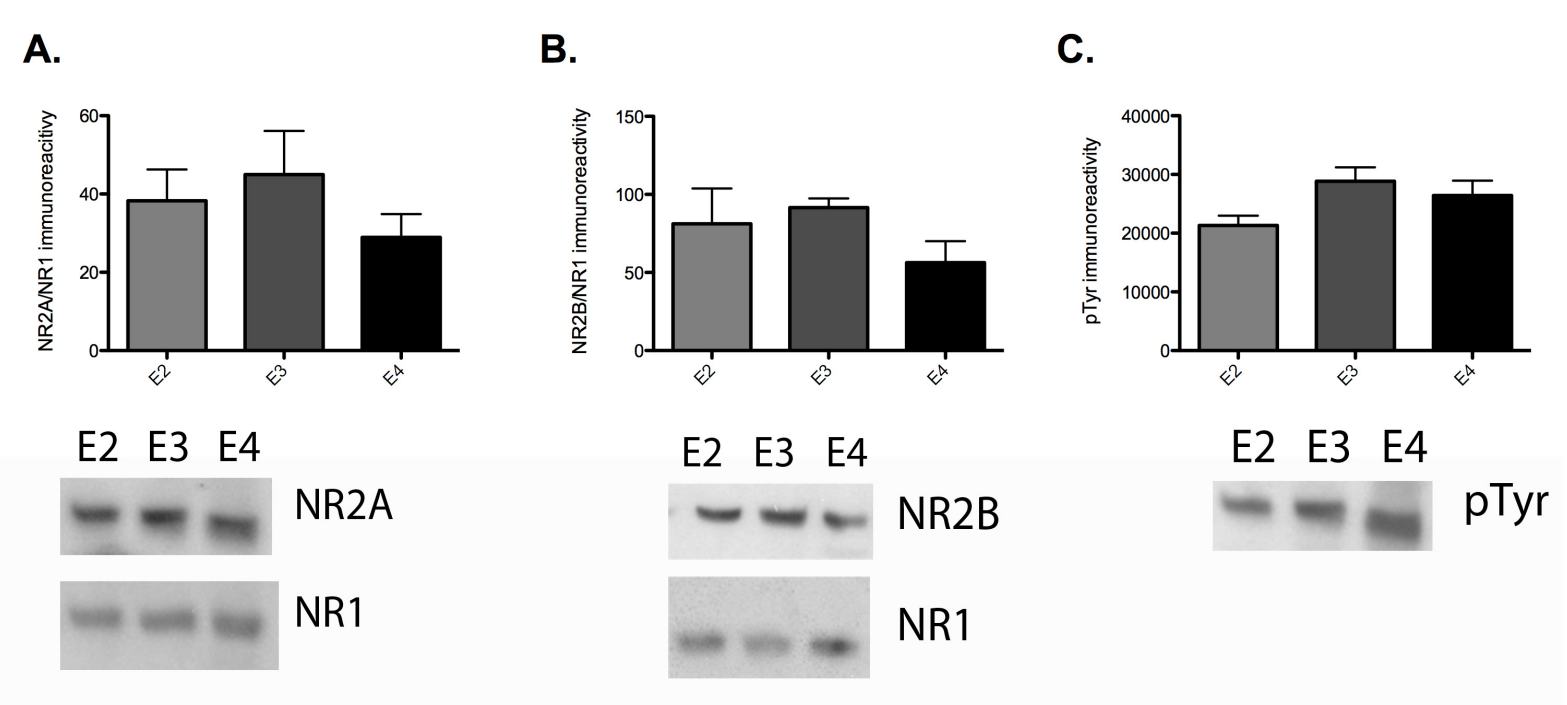
**Effect of chronic apoE isoform expression on NR2A, NR2B levels and tyrosine phosphorylation**. A) Levels of NR2A immunoreactivity, standardized to NR1 immunoreactivity, from CA1 of apoE TR animals. B) Levels of NR2B immunoreactivity, standardized to NR1 immunoreactivity, from CA1 of apoE TR animals. C) Levels of phosphotyrosine immunoreactivity at the molecular weight corresponding to NR2A and NR2B from CA1 of apoE TR animals. apoE2 TR (E2, medium grey), apoE3 TR (E3, dark grey), apoE4 TR (E4, black). Data expressed as mean ± SEM.

To test this possibility, we first attempted to immunoprecipitate NR2A and NR2B from hippocampus area CA1 samples from apoE TR animals. We found that there were significant difficulties in our ability to immunoprecipitate equivalent amounts either NR2A or NR2B from the different apoE TR animals (data not shown). While this indicates that there may be structural differences or unidentified protein interactions with NMDA receptors that prevent complete immunoprecipitation due to the presence of apoE isoforms, it also hindered our ability to determine potential isoform-specific modulation of NR2A and NR2B phosphorylation. Therefore, we chose to probe for NR1, NR2A and NR2B in homogenates of hippocampus area CA1. We found that there were no significant differences in total NR2A or NR2B levels, standardized to NR1, between apoE2, apoE3 and apoE4 TR animals (figure 6A, B). We also probed for total tyrosine phosphorylation at the corresponding to NR2A and NR2B protein band. Tyrosine phosphorylation at this molecular weight was also unchanged between apoE TR animals (figure 6C).

**Figure 7:**
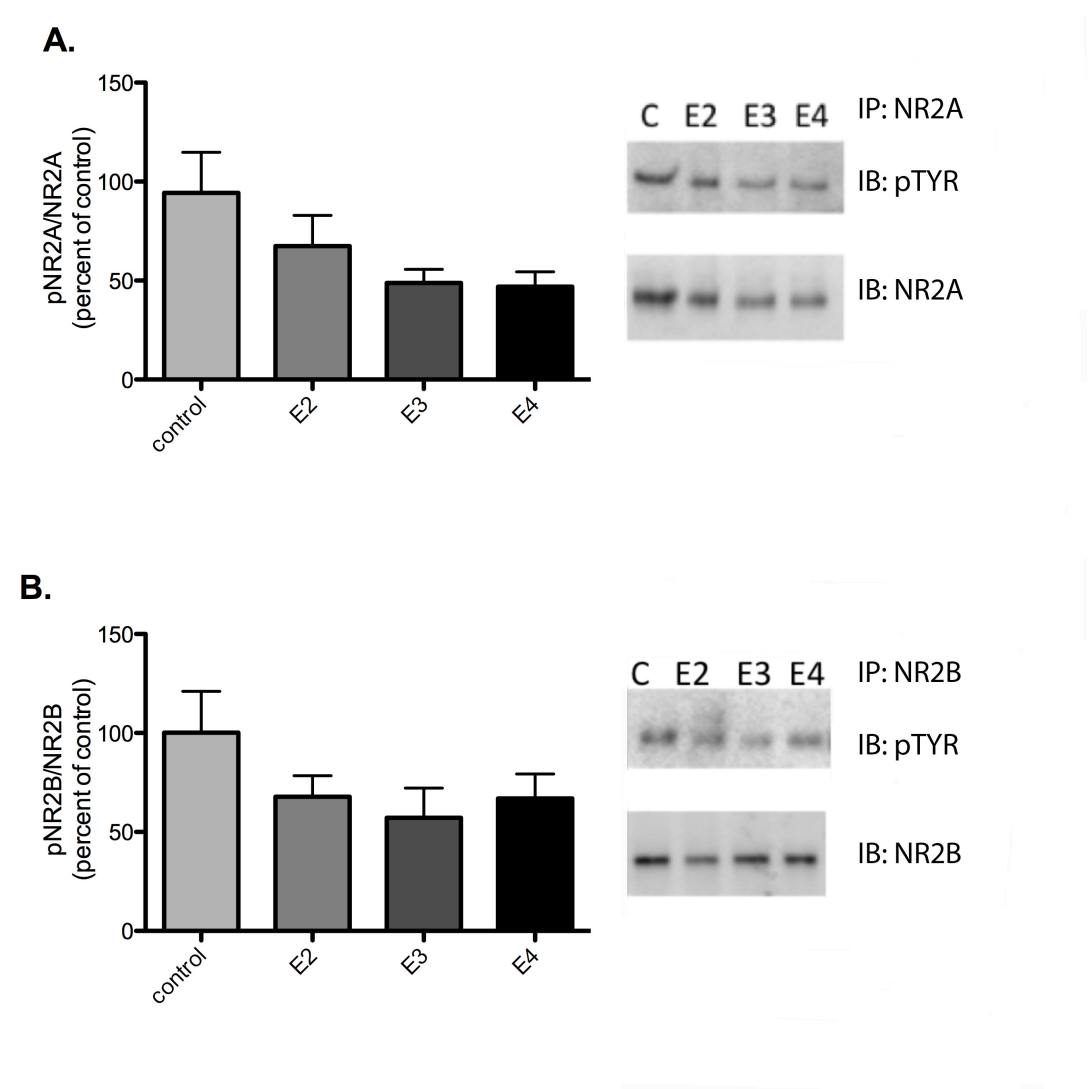
**Effect of acute apoE exposure on NR2A and NR2B tyrosine phosphorylation**. ApoE-deficient slices treated with 100 nM rhapoE isoforms. A) Ratio of pNR2A as measured by pTyr immunoreactivity to NR2A immunoreactivity as immunoprecipatated by NR2A. B) Ratio of pNR2B as measured by pTyr immunoreactivity to NR2B immunoreactivity as immunoprecipatated by NR2B. control (C, light grey), rhapoE2 (E2, medium grey), rhapoE3 (E3, dark grey), rhapoE4 (E4, black). Data expressed as mean ± SEM.

We were able to immunoprecipitate NR2A and NR2B from area CA1 of the hippocampus that had been treated with rhapoE isoforms. ApoE-deficient hippocampus slices were isolated and incubated with 100 nM recombinant apoE for 40 minutes before flash freezing on dry ice and rapidly dissecting out CA1. We found that there were no significant differences in the ratio of pNR2A to NR2A between any of the conditions, but there was a trend towards a reduced ratio in the rhapoE3 and rhapoE4 treated groups (figure 7A). Similarly, there were no significant differences in pNR2B to NR2B ratios (figure 7B).

### ApoE isoform alters signal transduction

ApoE has been previously shown in neuronal cell culture to have isoform specific effects on signal transduction pathways crucial to synaptic plasticity such as ERK and JNK [31]. As those effects were blocked with LDL receptor inhibitors and the NMDA receptor antagonist MK-801, we hypothesized that similar mechanisms may be underlying the isoform-specific alterations in CA1 LTP.

**Figure 8:**
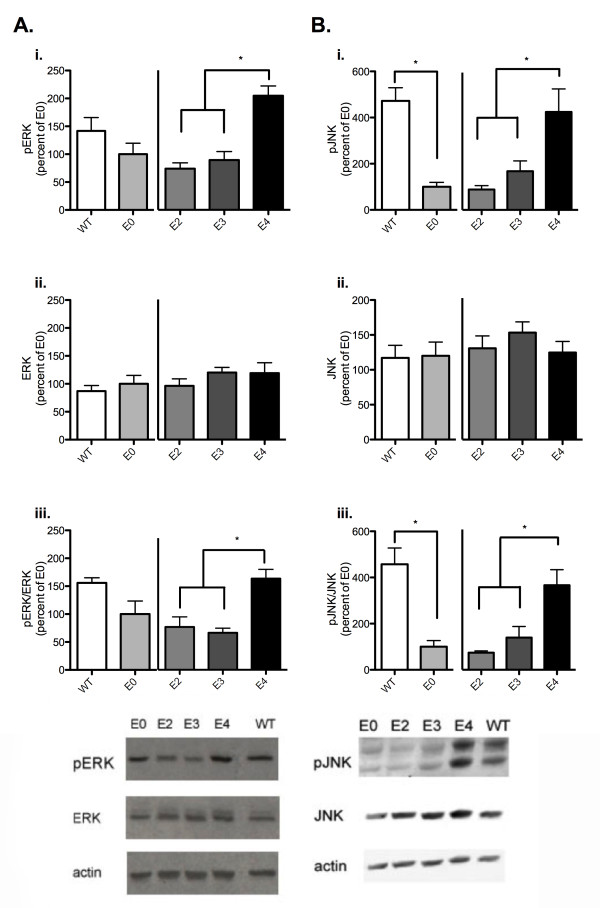
**Chronic apoE isoform expression alters activation of ERK1/2 and JNK1/2**. A) Quantification of levels of pERK (i) or ERK (ii) immunoreactivity in CA1 of apoE TR, wild-type, and apoE deficient animals. iii) pERK/ERK ratio. B) Quantification of levels of pJNK (i) or JNK (ii) immunoreactivity in CA1 of apoE TR, wild-type, or apoE deficient animals. iii) pJNK/JNK ratio. Quantification of immunoreactivity normalized to background (n = 5). C57BL/6J (WT, white), apoE-deficient (E0, light grey), apoE2 TR (E2, medium grey), apoE3 TR (E3, dark grey), apoE4 TR (E4, black). Data expressed as mean ± SEM. *p < 0.05, ANOVA with Bonferroni's posttest.

Area CA1 dissected from acute hippocampal slices of apoE TR, wild-type, and apoE-deficient animals were homogenized and subjected to SDS-PAGE analysis. Membranes were probed for pERK1/2, ERK1/2, pJNK1/2 or JNK1/2. This revealed that pERK1/2 was significantly increased in apoE4 TR animals versus apoE2 TR or apoE3 TR (figure 8Ai, ANOVA p = 0.0005). There were no accompanying significant changes in total ERK (figure 8Aii). The ratio of pERK to total ERK showed a significant increase in the apoE4 TR over apoE2 TR and apoE3 TR (figure 8Aiii, ANOVA p = 0.003).

When pJNK levels were analyzed, we found a significant reduction in apoE-deficient, versus wild-type animals (figure 8Bi, t-test p = 0.0003) and apoE2 and apoE3 TR versus apoE4 TR animals (figure 8Bi, ANOVA p = 0.0075). This was not accompanied by any significant changes in total JNK. The pJNK/JNK ratio revealed a significant reduction in apoE-deficient from wild-type (figure 8Bii, t-test 0.0015), and apoE2 TR and apoE3 TR animals from apoE4 TR (figure 8Biii, ANOVA p = 0.0025).

**Figure 9:**
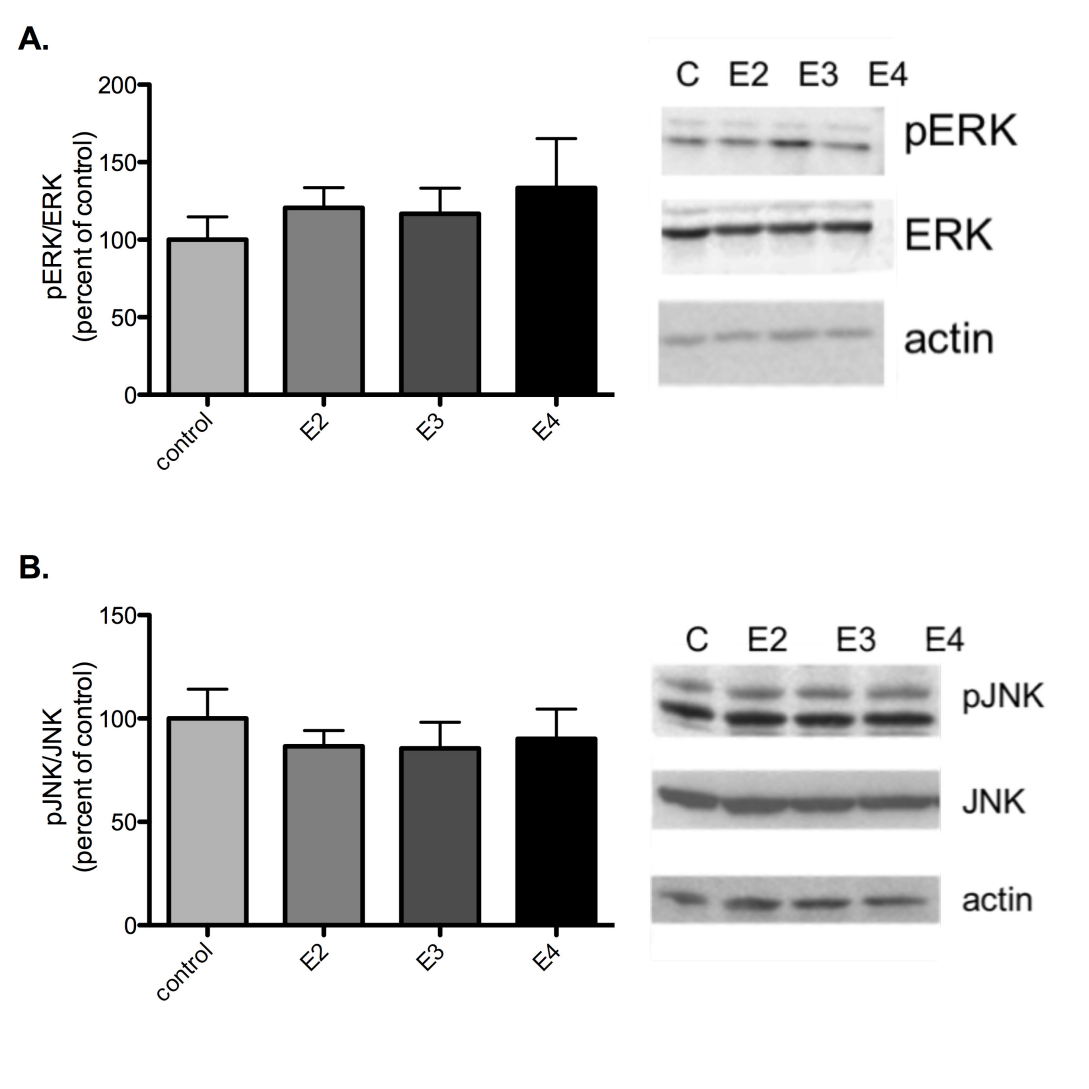
**Effects of acute apoE isoform exposure on ERK1/2 and JNK1/2 activation**. Quantification of A) pERK/ERK rato and B) pJNK/JNK ratio in CA1 of apoE-deficient animals treated with 100 nM rhapoE isoforms (n = 6 for each). Representative western blots showing levels of pERK, ERK, pJNK, JNK, and actin. control (C, light grey), rhapoE2 (E2, medium grey), rhapoE3 (E3, dark grey), rhapoE4 (E4, black). Data expressed as mean ± SEM.

In light of the differences in specific signal transduction proteins in the apoE TR mice, we wanted to test the effects of acute apoE exposure on signal transduction. ApoE-deficient hippocampus slices were isolated and incubated with 100 nM recombinant apoE for 40 minutes before flash freezing on dry ice and rapidly dissecting out CA1. Tissue samples were probed by western analysis for pERK, pJNK, ERK and JNK. Unlike comparable apoE TR mice, apoE isoform application did not significantly alter total pERK levels (data not shown). There were no significant differences in the pERK/ERK ratio (figure 9A). Furthermore, in contrast to what was seen with chronic apoE isoform expression, acute apoE isoform application did not significantly alter either JNK or pJNK levels (data not shown) or pJNK/JNK ratio (figure 9B). Taken together, these data suggest that the effects on signal transduction pathways and the potential compensatory changes of prolonged apoE isoform expression result in similar electrophysiology results, but may be more representative of different mechanisms underlying those physiologic effects.

## Discussion

Essential to defining how apoE influences the memory disruption and etiology of neurodegenerative disorders such as Alzheimer's disease is the establishment of the role of apoE in synaptic function. However, the physiologic actions of apoE as a signaling molecule in the adult CNS are unclear. In the present study we investigated both the necessity for murine apoE expression in adult hippocampus area CA1 synaptic plasticity as well as potential differences due to specific human apoE isoform expression. We found a lack of an overt physiologic defect in apoE-deficient animals with a C57Bl/6J background. This lack of phenotype was especially valuable in comparing differences with specific isoform expression in apoE TR mice. The apoE TR mice are particularly useful for these investigations as they allow for the direct *in vivo *comparison of the effects of apoE isoforms and reduce the potential caveats associated with apoE isoform over-expression. Importantly, in the absence of other genetic manipulations commonly used in mouse models of AD, these animals do not develop the potentially confounding pathological abnormalities know to affect memory formation and synaptic plasticity. Moreover, we were able to utilize the lack of a strong physiologic phenotype in apoE-deficient mice in conjunction with acute application of human recombinant apoE isoforms to preclude the potential confounding results of interactions or competition with endogenous murine apoE.

Previous electrophysiological studies of apoE TR mice have revealed that both perforant path LTP induction and the vulnerability of this potentiation by oligomeric beta amyloid (Aβ) vary with apoE isoform expression [15,19]. These studies show a decrease in LTP induction in apoE-deficient, apoE2 TR and apoE4 TR animals, with the greatest decrease in potentiation observed in apoE4 TR [15]. Our current study focuses on the well-characterized Schaffer collateral synapses, as does the previous work on reelin and its effects through the lipoprotein receptors apoER2 and VLDLR [9]. Although we predicted that the LTP induction profile would mirror that in the dentate gyrus, it was surprising to see that apoE4 TR mice demonstrated selective enhancement of CA1 LTP and apoE2 TR mice show the least amount of LTP induction.

ApoE isoforms acting as signaling ligands can change hippocampal physiology and alter LTP induction in a number of ways. One possibility is alteration of NMDA receptors through changes in subunit composition and/or phosphorylation. This possibility is already established as an action of reelin through apoER2 and VLDLR [21,22,32,33]. Our attempts to monitor changes in NMDAR subunit composition and phosphorylation through immunoprecipation revealed differences in our ability to pull down either NR2A or NR2B consistently between the apoE TR mice. Interestingly, total NR1 levels in these assays did not vary with NR2A or NR2B levels. We hypothesize that our ability to immunoprecipitate NR2A and NR2B is being hindered by differential treatment of NMDA receptors within neurons due to apoE signaling and our experimental conditions are not accurately controlling for these differences.

**Figure 10:**
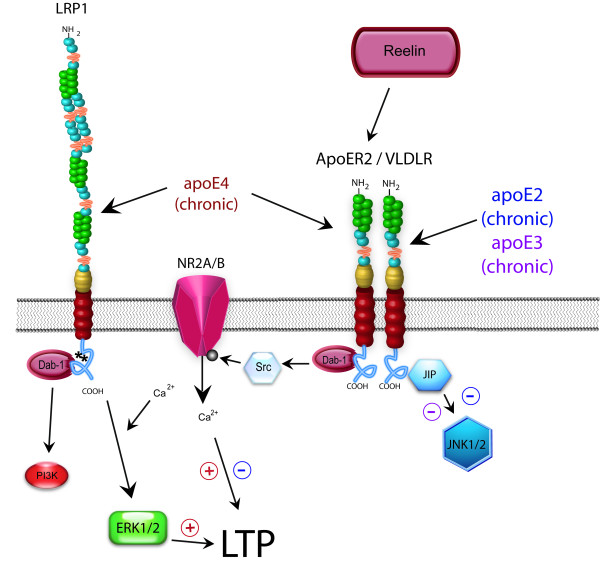
**Model of chronic apoE signaling in adult hippocampus**. Reelin interacts exclusively with the lipoprotein receptors apoER2 and VLDLR, with a much higher affinity for apoER2. ApoE binds to all lipoprotein receptors and undergoes endocytosis. Chronic apoE4 exposure enhances ERK1/2 activation, likely through interactions with the LRP1 receptor. In contrast, chronic apoE2 and apoE3 expression reduce activation of JNK1/2 and ERK1/2 activation. Together with proper NMDAR function, these changes culminate in alterations in LTP induction with chronic apoE2 isoform expression.

For the current study, we looked at total tyrosine phosphorylation corresponding to NR2A and NR2B in hippocampus area CA1 samples from apoE TR animals. We did not find any differences in total levels of NR2A, NR2B, or NR1 between groups; total tyrosine phosphorylation corresponding to NR2A and NR2B was also unchanged. These results indicate that within the total denatured hippocampal tissue our ability to detect NMDAR subunits are unhindered and no significant exists with the presence of specific apoE isoforms. However, it is difficult to discern whether specific changes in signal transduction pathways underlie the differences in LTP induction, or if there are chronic changes in signaling resulting in slight alterations to NMDAR subunit composition, localization or function that may cumulatively exert a physiologic effect on LTP. As shown by figure 10, it is likely that alterations in signal transduction via apoER2 that impact NMDA receptor function plus alterations in other signal transduction cascades combine to produce apoE isoform-specific changes in synaptic plasticity. Ongoing research in our laboratory is investigating these potential possibilities.

The changes in LTP induction with acute apoE isoform application are more difficult to explain. In this instance changes in NMDAR subunit composition are unlikely, and there were no significant changes in the ERK or JNK signal transduction pathways. Interestingly, rhapoE4 did not increase isolated NMDA receptor field potentials as expected, but rather caused a significant decrease. Yet there were no significant differences in the pNR2A/NR2A or pNR2B/NR2B ratios with treatment of any rhapoE isoform. While the observed trend towards decreased NR2A and NR2B phosphorylation with rhapoE4 treatment may actually contribute to the apoE-induced decreases in NMDAR field potentials, further studies will be necessary to determine if there are other mechanisms such as desensitization or internalization of NMDARs at work. Another possibility is that application of a bolus of apoE isoform selectively blocks lipoprotein receptor activity. This situation occurs with an antibody that binds to the reelin association site of apoER2 (CR-50), which results in a decrease in NMDA receptor whole cell currents [34]. Thus, the decrease in NMDA receptor currents with rhapoE2 and rhapoE4 may reflect an isoform-specific affinity for apoER2 when using non-cholesterol associated human recombinant protein. However, the ability for rhapoE4 to increase LTP induction despite these decreases in NMDAR field potentials suggests that apoE isoforms may also be acting differentially as signaling molecules to alter other components of the LTP machinery.

The idea of apoE as a signaling molecule is not new. In primary neuronal cell culture, application of either full length apoE or a tandem repeat peptide of the receptor binding domain of apoE significantly enhances phosphorylation of ERK1/2 and Dab-1 [32]. This pathway is well validated as essential to the underlying mechanisms of LTP [35,36], as well as learning and memory [37-39]. Modulation of these pathways by either pharmacological or genetic manipulation has dramatic effects on both synaptic plasticity and memory formation. With this in mind, an important aspect of these studies that should not be overlooked is the lack of an LTP phenotype in the apoE knockout mouse. This raises an interesting question: if apoE is acting as a signaling molecule important for neuronal function, then why is there no overt change in physiology in its absence? Our results suggest a unique role for specific apoE isoforms in the modulation of hippocampal CA1 synaptic plasticity. In addition, both apoE isoforms and reelin can bind to the same family of lipoprotein receptors. The role of reelin in synaptic function and memory formation is now well established, and specific isoform expression can have a direct effect on reelin signaling and modulation of synaptic function. Thus, apoE isoforms may function by changing reelin's ability to modulate NMDA receptors and activate specific signal transduction pathways by as yet unknown mechanisms. Furthermore, changes in apoE availability, due to spatial changes or associations with other proteins, may have a profound affect on synaptic function in the different subfields of the hippocampus.

With renewed focus on the role of apoE in neurodegenerative disorders and as a potentially rich area for therapeutic intervention comes the necessity to better understand how apoE can affect synaptic function. Evolution appears to have co-opted apoE to perform duties encompassing far more than cholesterol transport. Acting directly through lipoprotein receptor activation, or indirectly by altering reelin action, apoE isoforms can have a significant effect on NMDA receptor function. How might changes in apoE during normal and pathological states of neurodegeneration affect synaptic plasticity and memory formation? While this question is the foundation for future studies, our results here highlight the previously unappreciated role of apoE as a modulator of hippocampus synaptic plasticity.

## Experimental procedures

### Animal maintenance

ApoE2, apoE3 and apoE4 targeted replacement animals were obtained from a colony maintained at Taconic (Hudson, New York USA). ApoE-deficient and C57BL/6J animals were obtained from Jackson Laboratories (Bar Harbor, Maine, USA). Animals were housed in a standard 12 hour light cycle and bred and maintained in accordance with the Vanderbilt University Institutional Animal Care and Use Committee protocol.

### Electrophysiology

Hippocampus slices were prepared from 3- to 5-month old mice as previously reported [9]. The brain was rapidly removed and placed in oxygenated ice-cold high sucrose cutting saline solution containing (in mM) 110 sucrose, 60 NaCl, 3 KCl, 28 NaHCO_3_, 1.25 NaH_2_PO_4_, 5 glucose, 0.6 ascorbate, 7 MgCl_2_, and 0.5 CaCl_2_. Horizontal 400 μm sections were cut in high sucrose cutting solution using a vibratome. Slices were maintained in cold, oxygenated cutting solution until dissection. After dissection, the hippocampus slices were transferred to room temperature cutting solution diluted 1:1 with artificial cerebral spinal fluid (ACSF). ACSF contains, in mM, 125 NaCl, 2.5 KCl, 26 NaHCO_3_, 1.25 NaH_2_PO_4_, 25 glucose, 1 MgCl_2_, and 2 CaCl_2_. Slices were maintained in this solution with constant 95% O_2_/5% CO_2 _perfusion for 10 min before transferring to the brain slice recording chamber (Fine Science Tools, San Francisco, California, USA).

Extracellular recording was performed using standard techniques as previously reported [9]. The recording chamber was maintained at 30° ± 0.5°C with a laminar ACSF flow rate of approximately 1 mL/min. Field excitatory postsynaptic potentials (fEPSPs) were recorded from area CA1 *stratum radiatum *via glass micropipettes pulled to an approximate 1 μm tip diameter (1–4 MΩ) and filled with ACSF. Responses were generated by stimulation of fibers arising from the CA3 region. Stimulating electrodes consisting of formvar-coated nichrome wire delivered biphasic stimulus pulses (1–15 V, 100 μsec duration, 0.05 Hz). Delivery of stimulation, controlled by pClamp 9.0 software (Axon Instruments, Forster City, California, USA), was via the Digidata 1322A interface (Axon Instruments) and a stimulus isolator (model 2200, A-M Systems, Sequim, Washington, USA). Signals were amplified using a differential amplifier (model 1800, A-M Systems), filtered at 1 kHz and digitized at 10 kHz. For all experiments, baseline stimulus intensity was set at the level that elicited 40–50% of the maximum fEPSP response as determined from the input-output curve.

Long-term potentiation (LTP) was induced by theta-burst protocol. Theta-burst LTP protocol consisted of five trains of 10 bursts at a 5 Hz frequency with each burst consisting of 4 stimulations delivered at 100 Hz and an inter-train interval of 20 seconds. NMDAR-independent LTP was induced by 2 one-second trains of stimulation delivered at 200 Hz; concurrent application of 100 μM 2-amino-5-phosphonovaleric acid (APV) confirmed NMDAR independence. NMDAR field potentials were isolated by incubating slices on the rig in ACSF containing no Mg^2+ ^for 15 minutes followed by addition of 20 μM 6-cyano-7-nitroquinoxaline-2,3-dione (CNQX) and 25 μM picrotoxin. After 15 minutes of stabilization, slices were treated with 100 nM of recombinant human apoE isoforms (Calbiochem).

### Statistics

Electrophysiological data was also analyzed using one-way ANOVA with Bonferroni's post hoc tests. Significance was set at p < 0.05 for all tests.

### Biochemistry

#### ApoE/apoER2

Bilateral dissections of the whole hippocampus from aged (1 year old) animals were performed. Brain tissue was rapidly dissected and flash frozen on dry ice. Tissue was homogenized in NP-40 lysis buffer containing (in mM) 50 Tris-HCL ph 8.0, 150 NaCl, 1 EDTA, 1 PMSF, 1 Na_3_VO_4_, 1 NaF, 1 μg/mL each of aprotinin, leupeptin and pepstatin, and 1% NP-40. Protein concentration was determined by Bradford Assay (Bio-rad). For western blot analysis, 10 μg of protein was resolved by 4–20% gradient SDS-PAGE. The proteins were then transferred to PVDF membranes. Membranes were probed with goat anti-human apoE (Academy Bio-medical, Houston Texas, USA), rabbit anti-apoER2 (a gift of Drs. Gary Olson and Ray Burk, Vanderbilt University [40]), and rabbit anti-actin (Sigma) diluted in 0.24% I-block. Membranes were developed using HRP-conjugated secondary antibodies and enhanced chemiluminescence. Optical density of immunoreactivity was quantified by densitometry using Image J software (NIH).

For investigations of chronic apoE exposure, slices were obtained from apoE2 TR, apoE3 TR, apoE4 TR, apoE-deficient and C57BL/6J (wild-type) mice in an identical fashion as for electrophysiology (see above). The slices were frozen on dry ice and CA1 dissected unless otherwise stated. For investigations of acute apoE exposure, slices were obtained from apoE-deficient mice in an identical fashion as for electrophysiology (see above). Slices (n = 3–4 per treatment) were incubated in ACSF at 30°C for 1 hour prior to the addition of 100 nM recombinant human apoE2, apoE3 or apoE4 (Calbiochem) for 40 minutes. Slices were then flash frozen on dry ice and CA1 dissected.

#### ERK/JNK activation

Pooled tissue was homogenized in NP-40 lysis buffer containing (in mM) 50 Tris-HCL ph 8.0, 150 NaCl, 1 EDTA, 1 PMSF, 1 Na_3_VO_4_, 1 NaF, 1 μg/mL each of aprotinin, leupeptin and pepstatin, and 1% NP-40. Protein concentration was determined by Bradford Assay (Bio-Rad). Ten μg of protein was resolved by SDS-PAGE on 4–15% Tris-HCL gradient gels (Bio-Rad) and transferred to PVDF membrane. Membranes were probed with rabbit anti-ERK1/2, anti-ERK1/2 pTpY^185/187^, anti-JNK1/2, anti-JNK1/2 pTpY^183/185 ^(Invitrogen) diluted in 0.24% I-block. Membranes were developed using HRP-conjugated secondary antibodies and enhanced chemiluminescence.

#### NMDAR subunit phosphorylation

Pooled tissue was sonicated in modified RIPA buffer (Tris/HCl pH 7.4, 2 mM EDTA, 150 mM NaCl, 0.1% SDS, 0.5% sodium deoxycholate, 1% triton X100, 1× phosphatase inhibitors I and II (Sigma), and 1× complete protease inhibitors (Sigma). Protein concentrations were determined by BCA Protein Assay (Bio-Rad). Ten μg of protein was resolved by SDS-PAGE on 4–15% Tris-HCL gradient gels (Bio-Rad) and transferred to PVDF membrane. Membranes were probed with mouse anti-phosphotyrosine, clone 4G10 (Millipore), rabbit anti-NR2A, rabbit anti-NR2B (Upstate), and mouse anti-NR1 (Millipore) diluted in 0.24% I-block. Membranes were developed using HRP-conjugated secondary antibodies and enhanced chemiluminescence.

For immunoprecipitation assays, a total of 400 μg of protein lysate was used to immunoprecipitate either rabbit anti-NR2A or rabbit anti-NR2B (Upstate) overnight at 4°C with agitation. Protein A/G magnetic beads (New England BioLabs) were added to each reaction (25 μl bead slurry/reaction) and samples were incubated for 2 hours at 4°C with agitation. Following three wash cycles, the protein was eluted with 1× Laemmli sample buffer separated by SDS-PAGE on 4–15% Tris-HCl gradient gels (Bio-Rad) and transferred to PVDF membranes. Membranes were probed with mouse anti-pTyr, clone 4G10 (Millipore), rabbit anti-NR2A, and rabbit anti-NR2B in 2% BSA-TBST. Membranes were developed using HRP-conjugated secondary antibodies and enhanced chemiluminescence.

Optical density of immunoreactivity was quantified by densitometry using Image J software (NIH).

## Competing interests

The authors declare that they have no competing interests.

## Authors' contributions

KK performed the electrophysiological and biochemical studies. JT performed the immunoprecipitation assays and aided in the biochemical studies. ML provided the apoE TR mice and aided in the conceptual analysis. PS developed the apoE TR mice and approved their use in this study. EW directed the design and coordination of this study. All authors read and approved the final manuscript.
